# Rachel Challice's Vow

**Published:** 1888-02-11

**Authors:** Lina Nevill

**Affiliations:** Author of "A Future on Trust," "Juno," &c.


### RACHEL CHALLICE'S VOW.

By LINA NEVILL, Author of "A Future on Trust," "Juno," &c.

### CHAPTER VII.—RENOUNCING ALL.

"In some other happier clime
I may lose my sorrow;
Other, brighter days may rise—
Though to-day my spirit sighs,
It may smile again to-morrow."
—*Barry Cornwall.*

WHEN Rachel Challice parted from her sister and her new brother-in-law on Bedd-gelert Bridge, it was with the fixed determination of never, if she could help it, seeing either of them again.

She wished to put far away from her all the associations of her past life, to sever herself so utterly from all connexion with the scenes, events, and people she had known in youth, as to try and believe that they had never existed—that all remembrance was but some unreal though vivid dream.

The past was over and done with. She was not necessary to anything or anybody in Bedd-gelert, Ryhd-ddu, or Aberelwyn. Esther was married to the man of her choice, and would require her love and devotion no more. David had proved that he no longer cared for her. She had not a single friend in all the three parishes whom it should hurt her to leave, or who would regret her absence. Clearly, then, the past and present had no claim upon her. It was to the future she must look. And that future—so she had determined—was to have no link whatever with the scenes she was now leaving. All was to be new, all fresh; no single thread was to be left to bind her to what she now shrank from in disgust and weariness.

She walked quickly back from Bedd-gelert to the old home on the Ryhd-ddu road—the old home which was to know her no more.

A new owner was taking possession when she reached the cottage, another pensioner of Lord Merthyn's; a widow woman, who had bought all the Challices' household goods at a valuation, and who was in a hurry to come into and settle down in her new abode.

The house looked different already, as Rachel unlatched the gate and walked up the little garden path. Rugs and mats were strewn about outside; brushes and pails obstructed her way; and a big, gaunt woman, with a black bonnet tilted over her head, was bustling about inside the cottage.

"Oh, you've come for your things, have you?" said Mrs. Howell, pausing for a moment in her vigorous dusting and sweeping, a voluntary work of supererogation on her part, for Rachel had left the house as clean and neat as a new pin.

"There they are; I've put them all ready for you by the door, and shall be rather glad to get them out of my way. I'm sorry I can't ask you to sit down, but there is more work before me now than I can get through before my son comes home to-night."

"I have no time to spare either to sit down, thank you, Mrs. Howell; I shall have to walk fast as it is, to catch the London train at Ryhd-ddu."

"Well, good-bye now, and a pleasant journey to you,' called out the new tenant, returning with redoubled energy to the task of polishing a chair, which already shone brightly and gave forth a smell suggestive of very recent beeswax and turpentine.

Rachel took up the modest little carpet-bag which contained her limited wardrobe, and walked away quickly from the house that had been her home for the last seven years.

She had no treasures to carry away and add to the bulk of her luggage—no writing-desk, no work-box, no miscellaneous store of ornamental knick-knacks, such as most young girls possess.

Such trifles are generally gifts from loving donors. Rachel had no one to love her, or give her presents, or press pretty things on her acceptance. All she possessed in that way were a few small reward books, won as school prizes, which she valued little as coming from teachers who had never understood or cared for her.

One treasure she had—something which, though she despised her own weakness, she could not summon courage to part with or destroy. It was a dried sprig of forget-me-not, of that large, turquoise-coloured sort that grows so luxuriantly in damp, marshy places. David had picked it one evening when they had been walking together, and given it to her. Like the heroine of the song, she had fastened it carelessly enough in her dress, but nevertheless had guarded it jealously, and when she got home had dried it carefully between the leaves of her Prayer-book. She had it with her now, still lying safely in the book: a little bit of sentimental folly which proved that, after all, in spite of her firmness of character, Rachel Challice was nothing more nor less than a weak, loving woman, like most of her sisters in this big world of ours.

Mists wrapped the summits of the mountains, and veiled their hoary heads, as the girl passed along the wild lonely road, Colwyn rushed angrily over the boulders in its bed, swollen and turbid from recent rains. The moor looked damp and dreary, a wide-spreading, dismal, brown tract, bounded by the barren hillsides, with their shrouded summits.

Rachel shivered as she walked quickly on; it was all so melancholy and desolate.

She would be glad, she thought, to leave it all behind with her past life, and never see it again. She never thought of such a thing as possible home-sickness. She was going to a great city, wherein lurked brilliant hopes and promises—those boundless possibilities which the young are ever seeing in each phase and change of life, until middle age comes, and damps the ardent hopes with which they set out on their race.

Yes; Rachel was going to London, to the city paved with golden stones; to the great goal of all ambitious, restless spirits; the spot where in joy or sorrow the world's fierce heart throbs fastest.

There was a distant cousin of her grandfather's who once had come down to Aberelwyn on a visit, and had been kind to "poor Esther's children." Rachel had written to her, asking if she would take her in for a short time, and put her in the way of earning her own living. Miss Anne Challice had written promptly in reply that she was willing to receive Rachel for a short visit, and that she had better come up to town immediately, as she knew of some employment she thought would suit her.

So Rachel only waited to see Esther safely married, and then she started off alone to begin her struggle with the great unknown world, and make a niche for herself in its already crowded fanes.

Miss Anne Challice had done well for herself, and liked to see others striving to do the same. She had never married, considering matrimony a bad speculation—a lottery which no wise woman would think of putting into, containing as it did so many blanks and so few prizes. She liked girls who did not marry either. So there were two points in Rachel's favour to start with.

Anne Challice kept a greengrocer's shop in one of the streets off Tottenham Court Road, and drove a small though brisk business. She did not care to extend her connexion. She had no one to provide for after her death; and as long as she made enough to live upon comfortably she was quite satisfied, and was glad to avoid the extra trouble and anxiety a larger business would have brought with it.

Rachel at length reached Ryhd-ddu station, tired and fagged with her walk along the muddy road carrying her carpet-bag. All the country by this time was hidden from view in a sea of grey rolling mist, and a thick drizzling rain was falling. So, with dripping skies above her, as though Nature were bidding her a tearful farewell, Rachel Challice set out on her journey towards that golden land of promise which she fondly believed was waiting for her in the future.

### CHAPTER VIII.—AN OLD FRIEND WITH A NEW FACE.

"Tenderness, long forgotten, now burst forth,
Like rain-drops from the summer sky."—*L. E. L.*

THE busy hum and roar of ceaseless traffic, the endless rattle of a great city, were in full swing one broiling day about the middle of June. It was hot everywhere—hot even in the country; hot in the city parks and gardens; hot in the streets; and certainly it was hot enough in one of the wards of a large London hospital; though every window was opened, and every blind lowered to let in the air, and shut out the penetrating rays of the level afternoon sun, which glinted in between cracks and crevices, and made dusty motes dance across the ward, while the shadows seemed darker by contrast.

The noise of the streets rose up, and came in through the open windows. The street cries penetrated the sultry, still air, and brought visions of home, work, and association with other comrades, to many a man and boy lying on a bed of pain in the hospital wards, bearing weeks of enforced idleness, in some cases perhaps to end in total incapacity for life. Still there were many who would go out strong and well to begin again the busy round of daily toil, and with their voices help to swell the noise and tumult of the crowded London thoroughfares. It was just tea-time, and the nurses were going quickly to and fro, attending to the wants of the thirsty patients, who eagerly waited their turn to be served.

There was one bed, rather in the corner, with a bright-faced lad of about twenty for occupant, a cradle guarding his broken leg from the weight of the bedclothes. One of the nurses was coming towards him with a tray.

"Make haste, nurse," he called out cheerily. "I am dying for my tea. I thought you had forgotten me."

"Not much fear of that!" returned the nurse, laughing. "You would take pretty good care such a thing should not happen. Why, you make more noise and want more attention than all the rest of the ward put together."

"Well, it doesn't pay to be too meek, you know. I have observed from experience that it is the surest way of getting overlooked. Those who don't ask don't want—eh, nurse?"

"Then you will never come to want so long as your tongue is left you," replied the nurse in brisk tones, setting down the tea and bread and butter by him as she spoke.

"Don't go," said the lad, as she began to move away. "You are always in such a hurry when you come near me. It is very cruel, for you know I can't manage for myself with this game leg."

"Nonsense! You have the use of your hands, I suppose. You only want an excuse to talk, and I have no time to listen to you, with others waiting for their tea."

"Then come back as soon as you have fed them," he entreated plaintively. "Remember," he added, with a merry twinkle in his black, beady eyes, "I am accustomed to a great deal of attention; I am the only son of a widowed mother, and mother is Miss Challice's greatest chum. They have tea with each other every other day regularly; and, goodness me! the muffins and shrimps I've seen those two old ladies get through between them! I wonder the price doesn't go up on the spot."

"You are jealous because they do not invite you," replied the nurse, lingering in spite of herself.

The boy was so bright and good-tempered; he bore his pain and tedious confinement so bravely and patiently, always making the best of things, never repining or murmuring. No wonder everyone in the hospital who came in contact with him combined to spoil him.

"Oh, no, the grapes are not sour," he said quickly. "Miss Challice is quite devoted to me, and is always asking me to join them, only I am too shy to accept. To tell you the truth," he added confidentially, lowering his tone to a stage whisper, "I believe the old girl is madly in love with me. There are only twenty-five years difference in our ages; and that is nothing in these days, when every man marries his grandmother. And I always like to keep up with the fashion."

"You are a very impudent boy, and I will tell Miss Challice what you say."

"And I'll tell mother next time she comes to see me that you don't nurse me properly. And she'll tell Miss Challice; and won't you get a wigging next time you go to Percival Street! No tea and muffins for you next Sunday. No——"

But nurse was gone, and was attending to another patient at the further end of the ward.

"Whose voice is that above everyone else's?" asked the Sister of the ward, coming in at that moment. "Number Twelve as usual, I suppose. We shall have to muffle your tongue if you cannot keep it quiet," she added with a smile. "Now, can you tell me if Nurse Challice is here?"

"She is over there, at the other end," replied Number Twelve, perfectly unabashed at the rebuke.

He had earned for himself during his stay in the hospital the reputation of being a "character." This is a designation which usually means that the person so denominated is privileged to say whatever he chooses, rude or otherwise; to break rules with impunity; and have a free licence to habitually comport himself just as seems best in his own eyes. A "character" in these modern days is but the lineal descendant of the "fool" of our ancestors' era, minus the cap and bells and insignia of office.

"Nurse Challice," repeated the Sister; and the nurse who had given irrepressible Number Twelve his tea stepped forward at the call.

There she stood—Rachel Challice—with the same pure, pale, oval face; grey eyes, with long, shading black lashes; and dark hair, neatly smoothed away under the close nurse's cap.

The same girl—yet how different!

The old Rachel Challice had always worn a hard, weary look on her young face. Her manner always conveyed the impression that she was on the defensive, as though expecting to find an enemy in every person she met. Her voice had been cold and constrained, with little expression in its tones. The new Rachel Challice had the same features; but a different expression had crept over them. A calm, subdued gladness had come to take the place of discontent and weariness. Her voice had a cheerful ring in it, and a laugh was no uncommon thing to hear from her.

She had tried that panacea for all mental ills—real hard work—which, either selfishly or unselfishly undertaken, is such a royal road to health and happiness; and she had not found the recipe fail.

In looking after the ills and sorrows of others she found little time to remember and brood over her own. The wound was there still. The scar would never wholly disappear. The total forgetfulness which she had fondly imagined would come to her when once she had left behind her the scene of her youthful sorrows was not yet hers. But she could press now on sores where, before, the slightest touch made her wince, and cry out in inward agony. The first sharp sting had vanished. To forget, even partially, is human—the lawful, merciful birthright of our finite capacities.

For three years she had been working both in the hospital and on private nursing connected with the institution, and found the occupation exactly what she wanted. She had plenty of nerve; she loved the life; she was devoted to her patients, and they in return adored her.

For the first time in her nearly thirty years of life she found herself appreciated. And the good qualities of her nature, which before had been buried deep down, and never had the chance of springing up and showing themselves, bloomed forth in the new genial atmosphere, giving fair promise of a rich after-math.

In all these years she had heard nothing of her sister. She herself had never written, and Esther showed no signs of wishing to begin the correspondence. She knew absolutely nothing of how things were going on in Aber-elwyn. She had carried out her resolve to its furthest limit, and cut herself utterly adrift from all her old ties and associations, with no intention of ever again identifying herself with them.

She now came towards the Sister, in response to her call, and waited to hear what was wanted.

"The Matron wishes to speak to you, Nurse Challice. Can you go to her directly?"

Rachel replied in the affirmative; and wondering what could be wanted of her, went at once to the Matron's room.

She found that lady sitting before her writing-table, surrounded with letters and papers; an army of speaking-tubes bristled near, and one or two people were waiting in the anteroom for an interview.

"Who is that? Nurse Challice," she said, raising her head, and taking an envelope from a pile heaped beside her, "I want to send you to a private case. Nurse Wilkinson will be home to-night, so she can take her own work in the ward and leave you free."

She glanced over the letter she held, and went on: "The case is in Grosvenor Square—a young lady who was thrown from her horse. They have gone on without a regular nurse for some time; but the lady's maid is so knocked up—and not efficient either, they say—that they wish for a regularly trained person to take charge of Miss Endsleigh. You had better go to-morrow morning. There is the address—No. 100, Grosvenor Square."

Rachel took the envelope and left the room.

(*To be continued.*)

---

It is praiseworthy and necessary that all workers should join together to succour children, the aged, the sick, all those who cannot earn their bread by working for themselves. Doubtless the day will come when individual foresight and thrift will render hospitals and almshouses useless; but till then public benevolence and private charity have a noble task to perform.—*Edmond About.*

